# Sylvian Fissure and Parietal Anatomy in Children with Autism Spectrum Disorder

**DOI:** 10.3233/BEN-2012-110214

**Published:** 2012-02-15

**Authors:** Tracey A. Knaus, Helen Tager-Flusberg, Anne L. Foundas

**Affiliations:** ^1^Brain and Behavior Program at Children's HospitalLouisiana State University Health Sciences CenterNew OrleansLAUSA; ^2^Department of NeurologyLouisiana State University Health Sciences CenterNew OrleansLAUSA; ^3^Department of Cell Biology and AnatomyLouisiana State University Health Sciences CenterNew OrleansLAUSA; ^4^Department of PsychologyBoston UniversityBostonMAUSA

**Keywords:** Autism, parietal, Sylvian fissure, MRI, language, asymmetry

## Abstract

Autism spectrum disorder (ASD) is characterized by deficits in social functioning and language and communication, with restricted interests or stereotyped behaviors. Anatomical differences have been found in the parietal cortex in children with ASD, but parietal subregions and associations between Sylvian fissure (SF) and parietal anatomy have not been explored. In this study, SF length and anterior and posterior parietal volumes were measured on MRI in 30 right-handed boys with ASD and 30 right-handed typically developing boys (7–14 years), matched on age and non-verbal IQ. There was leftward SF and anterior parietal asymmetry, and rightward posterior parietal asymmetry, across groups. There were associations between SF and parietal asymmetries, with slight group differences. Typical SF asymmetry was associated with typical anterior and posterior parietal asymmetry, in both groups. In the atypical SF asymmetry group, controls had atypical parietal asymmetry, whereas in ASD there were more equal numbers of individuals with typical as atypical anterior parietal asymmetry. We did not find significant anatomical-behavioral associations. Our findings of more individuals in the ASD group having a dissociation between cortical asymmetries warrants further investigation of these subgroups and emphasizes the importance of investigating anatomical relationships in addition to group differences in individual regions.

## 1. Introduction

Both lesion and neuroimaging studies have shown that the left inferior parietal lobule (supramarginal gyrus, Brodmann area, BA 40) plays a critical role in the representation of learned, skilled movements (i.e. praxis representations) (for review [1]). Both action production and action understanding are thought to relate to language development and understanding of speech and gestures [2]. Action understanding and production are the building blocks of language, allowing communication between a sender and receiver [3] Planning tool-use actions has been shown to activate the same regions, including left parietal cortex, as planning communicative gestures [4]. Action production involves a distributed network that includes the anterior intraparietal cortex, which has strong connections to the supplementary motor cortex that functions in action planning and motor preparation [2]. Action understanding involves the mirror neuron system (MNS) [2,5]. Mirror neurons have been found in the inferior frontal gyrus, as well as in parietal areas, namely the supramarginal gyrus, providing further support for the importance of the inferior parietal cortex in action production and understanding [6,7, for review, 8]. Theoretically, when we witness actions performed by another the informa-

ISSN 0953-4180/12/$27.50 © 2012 – IOS Press and the authors. All rights reserved tion is subjected to sensory processing of elemental sensory inputs (e.g. visual gesture or auditory verbal) onto higher order association areas (i.e. visual analysis of object representation). These representations in turn must be mapped onto conceptual systems for the individual to know what others are doing (i.e. knowledge of action). These independent, but related processes, must develop and function in an integrated and efficient way if the individual is to acquire a gestural and language communication system. The right parietal cortex is more critical than the left homologue in mediating spatial perception, including the direction of visually guided movements and in directing attention with reference to body schema (for review [9,10]).

Parietal regions develop in relation to the Sylvian fissure (SF), which is formed by the overgrowth of adjacent brain regions (i.e. opercula), including the inferior frontal, superior temporal, and inferior parietal lobes, and is anatomically asymmetric. The left SF is longer in its posterior horizontal extension compared to the right (i.e. a longer horizontal length), while the right SF is shorter and tends to angle up more sharply (i.e. longer vertical length) (for reviews [11,12]). This configuration is found in over 75% of adults and children and SF anatomy has been found to be related to handedness [13,14] and language abilities [15]. In addition, a study examining the radiographic correlate of the posterior extent of the Sylvian fossa found an association with language laterality based on Wada testing [16]. Several post-mortem studies have shown that the adjacent parietal operculum develops asymmetrically in relation to these prominent SF asymmetries. Rubens and colleagues found an increase in the parietal operculum, rostral to the posterior ascending ramus in the left hemisphere (associated with the greater horizontal SF length) and an expansion caudal to the posterior ascending ramus in the right cerebral hemisphere (associated with the shortened horizontal SF length) [17]. A more recent MRI study of healthy adults showed the same relationships with the anterior and posterior segments of the parietal cortex linked to the SF anatomy [11]. These gross anatomical asymmetries may relate to hemispheric differences in parietal lobe functions.

Autism spectrum disorder (ASD) is an umbrella term, including classic autism, pervasive developmental disorder – not otherwise specified (PDD-NOS), and Asperger syndrome. ASD is a neurodevelopmental disorder defined by social deficits, language and communication impairments, and the presence of restricted or unusual interests or stereotyped behaviors [18]. Deficits in language and communication are a core feature of ASD, however, a range of language abilities is seen in ASD, from those who never develop functional language, to those who score normal or above on standardized language tests [19]. Subtle language problems exist in all individuals with ASD, particularly pragmatic or social aspects of language, non-literal interpretation, and semantic processing [20]. Although not part of the diagnostic criteria, motor deficits have consistently been found in individuals with ASD, including impairments in imitation (for review [21]) and in praxis functions [22]. Differences in motor planning [23,24] and difficulties in action understanding [25] have also been found in people with ASD. Some studies have reported deficits of the MNS in ASD (for review [26]) and although still unresolved [27,28], several groups have proposed that a dysfunction in the MNS is responsible for core social and language deficits in ASD (i.e. the broken mirror neuron theory of ASD) (for review [26, 29]). Given the importance of the parietal cortex in action planning and understanding and in the MNS, these behavioral findings suggest that there may be dysfunction within these neural systems that could be found at the level of macroscopic anatomical anomalies in the parietal region.

Several recent studies have examined the anatomy of the parietal lobe in ASD, with some differences being noted. A larger parietal volume has been found in children, after controlling for total brain volume [30], and in adolescents and young adults with ASD [31] relative to controls. Increased cortical thickness has been documented in parietal regions in children [32] and adolescents with ASD [33] compared to typically developing individuals. In contrast to these findings, one recent study found that the left parietal cortex was thinner in children and adults with ASD relative to controls with this thinner parietal cortex found bilaterally in children [34]. Differences in cortical folding have also been found in the parietal cortex in ASD. One study reported increased cortical folding in ASD relative to controls with a stronger effect in children compared to adolescents [35]. Another study investigated monozygotic twins discordant for ASD compared to typically developing controls and found increased cortical folding in the right parietal lobe in children with ASD and their unaffected co-twins relative to controls [36]. A study of young children found increased white matter in parietal regions in the ASD group compared to controls [37]. A recent study examined the relationship between parietal size and language and motor functions in children with ASD [38]. They reported a significant correlation between left parietal gray matter volume with age of first word, such that larger volume was associated with more delayed language. Differences in the anterior and posterior subregions of the parietal lobe and associations with current language measures have not been examined in ASD. In addition, anatomical relationships between cortical regions, such as associations between parietal subregions and SF anatomy have not been investigated in ASD, despite evidence of differential cortical development and the importance of these anatomical asymmetries in the development of lateralized language and praxis functions [39–41].

Few studies have examined SF anatomy in individuals with ASD. One study, however, by Levitt and colleagues [42] mapped the location of major cortical sulci in children with ASD and found an anterior and/or superior shift in location in frontal, temporal, and parietal regions in the ASD group relative to controls, suggesting that differences in the fissures and sulci that demarcate the adjacent opercular structures may be found in ASD. Findings included an anterior shift of the superior temporal sulcus (STS) and a superior shift of the intraparietal sulcus and ascending rami of the STS, bilaterally. This effect was larger in the left hemisphere, suggesting that temporal and parietal cortical regions in the left hemisphere may be more anatomically anomalous compared to these same regions in the right hemisphere. The SF was also shifted rostrally by about 6 mm in the right hemisphere in the ASD compared to control groups. In both groups, however, the SF extended more posteriorly in the left relative to the right hemisphere, consistent with the most typical SF configuration.

The first goal of this study was to develop a valid and reliable method for measuring the full extent of the anterior and posterior parietal subregions on MR images using anatomical landmarks to define functional-anatomical regions of interest (ROIs). The second goal was to examine the anatomy of these regions, as well as their relation to SF anatomy, in a group of children with ASD compared to a matched group of typically developing children, ages 7 to 14 years. Another goal was to determine whether scores on standardized language tests were associated with anatomical measurements of perisylvian anatomy. It was predicted that the typically developing children would have a longer left than right horizontal SF, leftward asymmetry of anterior parietal volume, and the posterior parietal volume would be expanded in the right hemisphere relative to the left, similar to what has been shown with length measurements on the cortical surface in healthy adults [11,17]. It was also postulated that there would be associations between SF anatomy and parietal volumes, based on previous studies in typical adults [11, 17]. In children with ASD, we hypothesized that the parietal anatomy would be atypical compared to controls. Specifically, it was postulated that there would be differences in anterior parietal anatomy, especially in the left hemisphere, based on findings that individuals with ASD have impairments in praxis [22] and motor planning [23,24]. Based on findings of impairments of the MNS in ASD (for review [26]), it was postulated that there would be differences in posterior parietal anatomy. It was also hypothesized that there would be more dissociations between SF and parietal anatomy in the ASD group [39–41]. Zoccante and colleagues [38] found an association between left parietal volume and language delay in children with ASD, leading us to further predict associations between language and anatomy in ASD, such that subjects with atypical anatomy would be more likely to have impaired language, based on standardized tests.

## 2. Material and methods

### 2.1. Subjects

Subjects included 30 boys with ASD and 30 typically developing boys, 7–14 years old. All subjects had English as their first language and were right-handed, based on scores from the modified Dean handedness inventory [43]. This inventory consists of 12 unimanual tasks, with scores ranging from $-24$, indicating complete left-handedness, to $+24$, indicating complete right-handedness, and all subjects had positive scores. Groups were matched on age and non-verbal IQ. Individuals with frank neurological damage, with a known genetic disorder, who were born prematurely (less than 35 weeks), or who had had seizures within the last 3 years were excluded from the study. Typically developing individuals also had no history or current diagnosis of developmental, learning, psychiatric, or neurologic disorders and no immediate family members with an ASD diagnosis.

For ASD participants, diagnosis was based on DSM-IV criteria [18] using the Autism Diagnostic Interview-Revised (ADI-R) [44] and the Autism Diagnostic Observation Schedule, Module 3 (ADOS) [45] administered by trained examiners. In addition, an expert clinician confirmed that all individuals met criteria for ASD. Individuals with ASD were high-functioning, with non-verbal IQ scores of 90 or higher.

Table 1
Subject characteristics

| | ASD | Controls |
|---|---|---|
| Age | 10.75 (2.12) | 10.79 (2.35) |
| Handedness | 16.83 (6.49) | 19.10 (4.56) |
| Verbal IQ | 103.77 (19.32) | 113.23 (13.55) |
| Non-verbal IQ | 109.60 (10.22) | 111.87 (11.05) |
| Full scale IQ | 108.03 (13.80) | 114.97 (11.89) |
| Receptive language | 101.69 (15.52) | 113.63 (16.67) |
| Expressive language | 97.48 (16.04) | 109.50 (12.83) |
| Total language | 99.66 (15.96) | 112.73 (13.92) |
| ADOS communication | 3.53 (1.36) | – |
| ADOS social | 9.27 (2.65) | – |
| ADOS total | 12.80 (3.51) | – |

Parents and participants were informed of the procedures and parents gave written consent prior to the child's participation in the study. Children 12 years and older also provided written assent prior to participation. All data in this manuscript were collected in compliance with the Boston University School of Medicine Institutional Review Board.

## 2.2. *Standardized tests*

The Kaufman Brief Intelligence Test (KBIT-II) [46] was administered to all subjects in order to assess IQ and the Clinical Evaluation of Language Fundamentals (CELF, 3rd ed) [47] was administered to assess receptive and expressive language. One subject did not complete language testing. For the CELF, 4 subtests were administered, which varied based on the age of the subject. For participants 7–8 years old, Concepts and Directions, Sentence Structure (receptive language), Recalling Sentences, and Word Structure (expressive language) subtests were given. For participants 9 years and older, Concepts and Directions, Word Classes (receptive language), Formulated Sentences, and Recalling Sentences (expressive language) subtests were administered. See Table 1 for subject characteristics.

## 2.3. *MRI acquisition*

All participants were trained in a mock scanner prior to the actual MR scanning. Volumetric MR images were acquired on a Philips 3 T Intera scanner. T1-weighted images were obtained as a series of 160, 1 mm gapless sagittal images. Turbo field echo (TFE) was used, with technical factors of: TR = 9.9 s, TE = 4.6, 256 × 256 pixel matrix, 24 cm field of view, and 8° flip angle. Data sets were rotated into alignment in the sagittal, axial, and coronal planes in order to eliminate any head rotation and MRI scans were maintained in real space. Each MRI scan series was assigned a blind number to assure subject confidentiality and to ensure that all measurements were performed blind to group and subject.

## 2.4. *MRI measurements*

### 2.4.1. *Total hemisphere volume*

Total hemisphere volume was measured with the MEASURE program [48]. The BET tool in FSL [49] was first used to remove as much skull as possible, without removing any brain. The contrast was then set and the MEASURE algorithm automatically outlined the brain. The midline image was identified and all outlining on this image was removed. This image was not included in the measure of left or right hemisphere volume. The outlining was then manually edited in each image to remove any remaining non-brain regions from the outline. This measurement included gray and white matter and the thalamus, but excluded the cerebellum and brainstem. The MEASURE algorithm calculated the volume in $cm^3$, based on the slice thickness and the area outlined on each image. For inter-rater reliability, a subset of five brains (ten hemispheres) was measured by four investigators. An intra-class correlation (ICC) was calculated between the investigators' volume measurements across both hemispheres of the five brains. The ICC for total brain volume was 0.97.

### 2.4.2. *Regions of interest*

Each ROI was measured in each hemisphere, using MRIcro [50]. Sagittal and axial views were primarily used. Boundaries were located and marked in the sagittal and coronal planes and the axial plane was used to measure the lateral portions of the regions. MRIcro algorithms calculated the number of voxels within the ROI. Length was calculated in cm based on voxel size and volume was calculated in $cm^3$ based on voxel size and slice thickness. Intra-rater reliability was established using a subset of 5 brains (10 hemispheres). ROIs were re-measured in these 5 brains and ICCs were calculated between the first measurements and the second measurements of each region, across both hemispheres.

#### 2.4.2.1. *Sylvian Fissure* (*SF*) *length*

The length of the horizontal SF was measured on three consecutive sagittal images in each hemisphere and the average length was calculated. The medial point was defined as the sagittal image where the SF begins to open into the Sylvian fossa, but prior to the

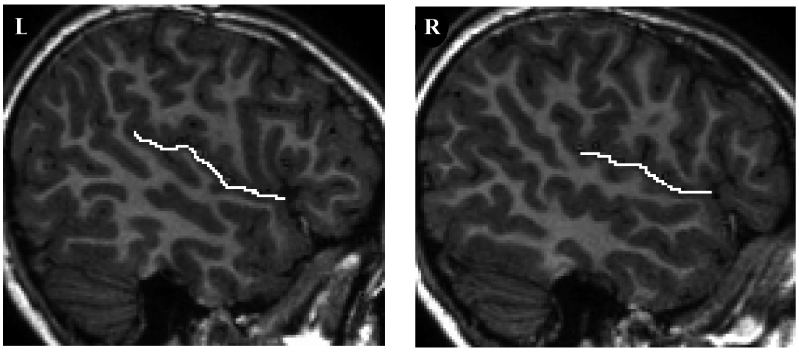

(a) (b)

Fig. 1. The Sylvian Fissure measurement in the sagittal plane in a.) the left hemisphere and b.) the right hemisphere of the same brain.

insula being visible. The sagittal image chosen was the slice that was 1/4 the distance from the medial point to the most lateral and 1 image on either side of this image was included in the measure. This is similar to the method used by Hellige et al. [51] and allows the Sylvian fissure to be measured at its longest horizontal extent. The anterior boundary was the point where the anterior horizontal and ascending rami bifurcate. In cases where the anterior ascending ramus branched off the anterior horizontal ramus, rather than the SF, the point where the anterior horizontal ramus turned upward was used. The posterior boundary was the end of the horizontal ramus, where it turns upward into the posterior ascending ramus. Refer to Fig. 1 for a view of this ROI. The ICC for intra-rater reliability was 0.83. There is much individual variability in this region, especially in the posterior portion of the SF, which can make reliable measurements difficult. Several distinct configurations were seen and defined as L-shaped (posterior ascending ramus only), T-shaped (bifurcation into posterior ascending and descending rami, with rami of similar size), and $T_a$-shaped (bifurcation into posterior ascending and descending rami, with descending ramus much smaller than ascending). Pseudo posterior ascending rami were also sometimes seen and the posterior ascending ramus was defined as the most anterior point of turn-up, posterior to the post-central gyrus. In some cases, the SF gently sloped upward, which can make determination of the posterior boundary difficult. In these cases, the sharpest turn-up point was determined and the superior temporal gyrus was also used to aid in finding this boundary, as it often follows a similar pattern as the SF. For unusual or difficult cases, investigators met to discuss and a consensus was reached. For our reliability, the same shape was generally measured, with some slight variability in the precise location of the posterior boundary, however, due to the small size of the measurement, extremely small differences caused a large impact on the ICC.

*2.4.2.2. Anterior parietal volume*

This region is located superior to the SF and caudal to the central sulcus as depicted in Fig. 2. This ROI is largely composed of the supramarginal gyrus (BA 40), postcentral sulcus, and posterior bank of the central sulcus. The anterior boundary was determined by Heschl's transverse gyrus, along the superior bank of the STS. This anterior boundary was defined in the coronal plane as the most anterior image in which Heschl's gyrus was clearly visible, with a small amount of white matter lateral to it. The posterior boundary was defined as the end of the horizontal ramus of the SF in the sagittal plane. The inferior boundary was demarcated by the horizontal SF and all cortex superior to this structure was included in the measure. The medial boundary was defined as the sagittal image where the SF begins to open into the Sylvian fossa, but prior to the insula being visible. The axial plane was used to measure the lateral edge, so that the entire cortex lateral to the medial boundary was included in the ROI. The intra-rater ICC for this region was 0.96.

*2.4.2.3. Posterior parietal volume*

This region is located superior to the horizontal SF and extends caudal to the anterior parietal ROI as depicted in Fig. 3. This ROI included the angular gyrus (BA 39), a posterior portion of the supramarginal gyrus, and portions of the precuneus. The anterior boundary was the same as the posterior boundary of the anterior parietal ROI (i.e. the end of the horizontal ramus of the SF). The entire cortical area located posterior to this vertical-anterior boundary was included in the ROI. The inferior boundary was defined by extending a straight horizontal line from the end of the horizon-

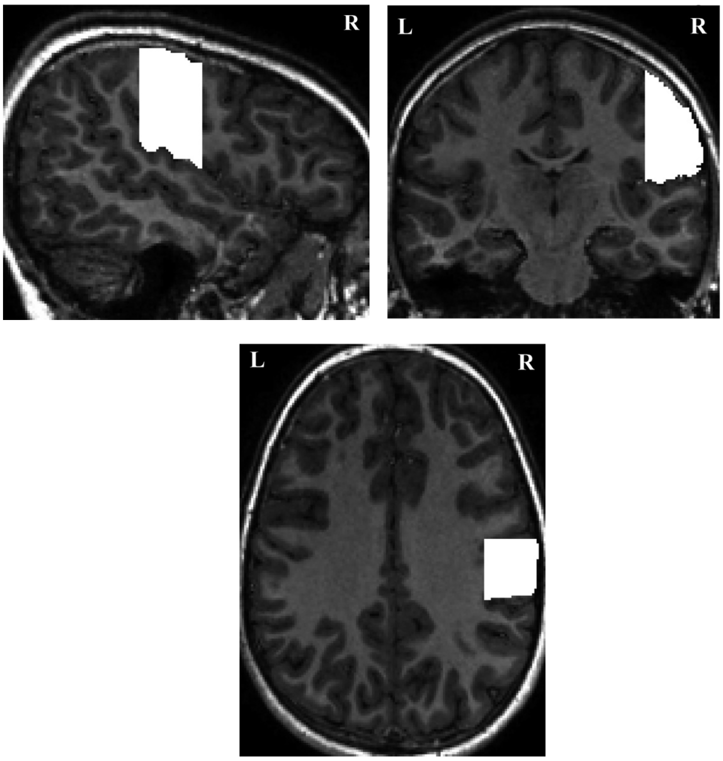

Fig. 2. The anterior parietal measurement in the sagittal, coronal, and axial planes.

tal ramus of the SF to the occiput. The same medial boundary was used as described for the anterior parietal ROI. The ICC for intra-rater reliability was 0.95.

## 2.5. *Analyses*

### 2.5.1. *Behavioral measures*

A multivariate analysis of variance (MANOVA) was used to examine differences in IQ between the ASD and control groups. Diagnosis (ASD, control) was the independent variable and K-BIT verbal and non-verbal standard scores were the dependent variables. Similarly, differences in language scores were examined via MANOVA, with diagnosis as the independent variable and CELF receptive and expressive standard scores as dependent variables. ANOVA was used to examine group differences in degree of handedness, with diagnosis as the independent variable and Dean handedness score as the dependent variable.

### 2.5.2. *Size and asymmetry*

To control for differences in total brain size, volumes were calculated as a proportion of total hemisphere volume and this proportion was used for all volume analyses. To examine differences between the ASD and control groups, MANOVA was used with hemisphere as the within-subjects independent variable, diagnosis (ASD, control) as the between-subjects independent variable, and posterior parietal volume, anterior parietal volume, and SF length as the dependent variables. An asymmetry quotient (AQ) was calculated for each region, as (L-R)/[(L+R)/2], so that a positive AQ indicated leftward asymmetry and a negative quotient indicated rightward asymmetry. AQs between $-0.025$ and $+0.025$ were considered symmetric. In order to examine group differences in degree of asymmetry, MANOVA was used, with diagnosis as the independent variable and posterior parietal, anterior parietal, and SF AQs as dependent variables. To examine differences in the direction of asymmetry, chi-squares were calculated for each region.

### 2.5.3. *Anatomical associations*

To examine associations between parietal anatomy and SF configuration, the sample was divided into those with typical, leftward SF asymmetry, and those with atypical (rightward or no asymmetry) asymmetry of the SF. MANOVA was used with hemisphere as the within-subjects independent variable, diagnosis (ASD, control) and SF (typical, atypical) as the between sub-

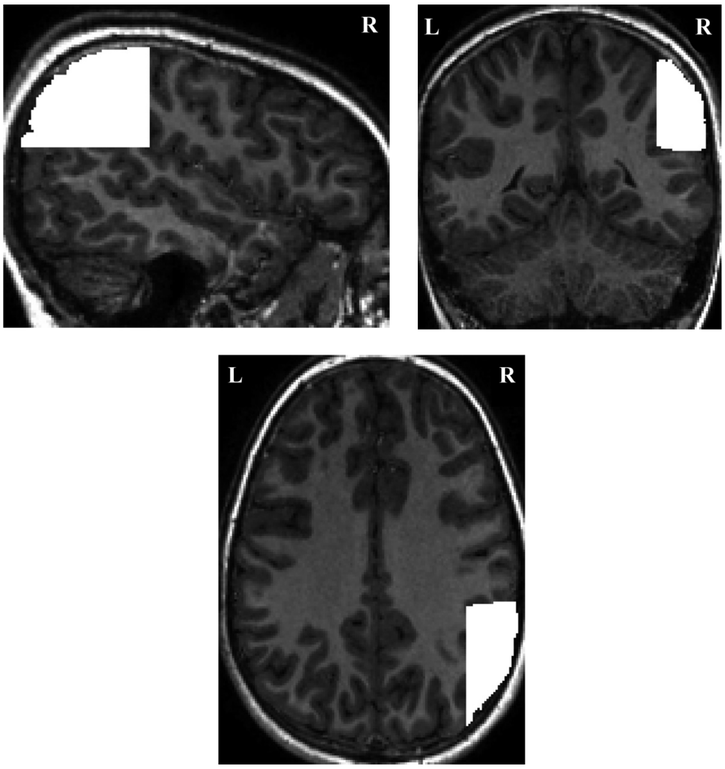

Fig. 3. The posterior parietal measurement in all 3 planes.

jects independent variables, and posterior and anterior parietal volumes as dependent variables. Similarly, to examine differences in parietal AQs, a MANOVA was calculated with diagnosis and SF configuration as the independent variables and posterior and anterior parietal AQs as dependent variables. Chi-squares were also performed in each diagnostic group separately to examine differences in the direction of asymmetry between typical and atypical SF asymmetry groups.

Pearson correlations were calculated in each group to examine anatomical relationships. Correlations between posterior and anterior parietal volumes, SF and posterior parietal volume, and SF and anterior parietal volume, in each hemisphere were performed. Associations between AQs were also examined for posterior and anterior parietal, SF and posterior parietal, and SF and anterior parietal.

*2.5.4. Relation to language*

Several methods were used to explore potential relationships between behavioral and anatomical measures. Pearson correlations between ROI size and asymmetry with verbal IQ, nonverbal IQ, and CELF receptive and expressive scores were examined in the ASD group.

The ASD sample was divided into those with low language and average/high language, based on standardized tests. Any subject with a score of 85 or less (below 1 standard deviation) on either the verbal IQ, receptive, or expressive language subtests was classified as low language and those with scores higher than 85 on all 3 language subtests were put in the average/high language group. To examine differences in volume between these groups, MANOVA was used with hemisphere as the within-subjects independent variable and language group (low, average/high) as the between subjects independent variable, and SF length and anterior and posterior parietal volumes as dependent variables. Similarly, to examine differences in asymmetry, MANOVA was performed with language group as the independent variable and SF and anterior and posterior parietal AQs as dependent variables.

The ASD sample was divided based on anatomical asymmetry; those with expected asymmetry of all 3 ROIs (L>R SF, L>R anterior parietal, R>L posterior parietal) were put in the expected anatomy group and those who had atypical asymmetry of any one of the ROIs were classified as unexpected anatomy. To compare these groups on behavioral measures, MANOVA was calculated with anatomical group (expected, unex-

Table 2
ROI and total hemisphere size and AQ

| | ASD | Controls |
|---|---|---|
| Left SF length | 0.7281 (0.1126) | 0.7302 (0.0781) |
| Right SF length | 0.6720 (0.0804) | 0.6602 (0.0801) |
| SF AQ | +0.0751 (0.1892) | +0.1016 (0.1375) |
| Left hemisphere volume | 671.02 (62.23) | 671.04 (52.37) |
| Right hemisphere volume | 676.74 (61.59) | 665.54 (54.98) |
| Left anterior parietal volume | 0.0302 (0.0066) | 0.0296 (0.0080) |
| Right anterior parietal volume | 0.0238 (0.0067) | 0.0259 (0.0069) |
| Anterior parietal AQ | +0.2477 (0.2635) | +0.1398 (0.3823) |
| Left posterior parietal volume | 0.0351 (0.0122) | 0.0349 (0.0108) |
| Right posterior parietal volume | 0.0380 (0.0099) | 0.0402 (0.0079) |
| Posterior parietal AQ | −0.1181 (0.4896) | −0.1591 (0.3461) |

Volumes are proportional to total hemisphere volume.

pected) as the independent variable and verbal IQ, receptive language, expressive language, ADOS communication, and ADOS social scores as dependent variables.

## 3. Results

### 3.1. Behavioral measures

For IQ, at the multivariate level, there were no significant group differences. For language, at the multivariate level, there was a significant main effect of group ($F_{2,56} = 5.78$, $p = 0.005$). At the univariate level, this was significant for both receptive ($F_{1,57} = 8.10$, $p = 0.006$) and expressive ($F_{1,57} = 10.14$, $p = 0.002$) language scores, with the typically developing group having significantly higher language scores than the ASD group. There were no significant group differences in degree of handedness. See Table 1 for means and standard deviations.

### 3.2. Size and asymmetry

At the multivariate level, there was a significant hemisphere effect ($F_{3,56} = 8.53$, $p < 0.001$), with no group or group by hemisphere effects. The univariate level indicated that SF length ($F_{1,58} = 16.75$, $p < 0.001$) and anterior parietal volume ($F_{1,58} = 19.08$, $p < 0.001$) were significantly larger in the left hemisphere compared to the right and that there was significantly larger right than left volume for the posterior parietal ($F_{1,58} = 5.53$, $p = 0.022$). There were no significant group differences in degree or direction of asymmetry for any region. See Table 2 for means and standard deviations.

### 3.3. Anatomical associations

The MANOVA examining associations with SF anatomy revealed a significant hemisphere by SF interaction ($F_{2,55} = 26.03$, $p < 0.001$). At the univariate level, this was significant for anterior ($F_{1,56} = 16.09$, $p < 0.001$) and posterior parietal volumes ($F_{1,56} = 42.02$, $p < 0.001$). For the anterior parietal ROI, we found that those individuals with typical SF asymmetry (leftward) had larger volume in the left than right hemisphere and those with atypical SF asymmetry did not demonstrate differences between the hemispheres. For the posterior region, those with typical SF asymmetry had larger right than left volume, while those with atypical SF asymmetry had larger volume in the left hemisphere compared to the right. When AQs were examined, we found a significant SF configuration effect ($F_{2,55} = 22.73$, $p < 0.001$), which was significant for both parietal regions (anterior $F_{1,56} = 14.09$, $p < 0.001$; posterior $F_{1,56} = 37.35$, $p < 0.001$). Both SF groups had leftward asymmetry of the anterior parietal ROI, however, the asymmetry was significantly stronger in the typical than atypical SF group. For the posterior parietal, those with typical SF asymmetry had rightward asymmetry and those with atypical SF asymmetry demonstrated leftward asymmetry. The chi-square for controls was significant for the anterior parietal region ($\chi^2 = 8.68, p = 0.013$), with more subjects having leftward asymmetry in the typical SF group and more having rightward asymmetry in the atypical SF group. The chi-square for ASD, however, was not significant ($\chi^2 = 2.94$, $p = 0.087$), with both SF groups having more individuals with leftward anterior parietal asymmetry (Fig. 4). For the posterior parietal, chi-squares were significant in both groups (ASD $\chi^2 = 13.03$, $p < 0.001$; controls $\chi^2 = 13.38$, $p = 0.001$). In the typical SF group, there were more subjects with

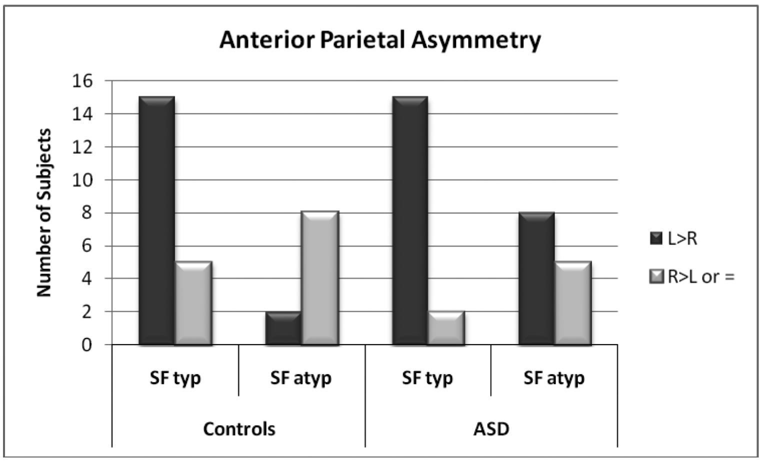

Fig. 4. The number of subjects with leftward asymmetry (typical) and rightward or no asymmetry of anterior parietal measures broken down by SF configuration (typical, L>R or atypical, R>L or =) and diagnostic group.

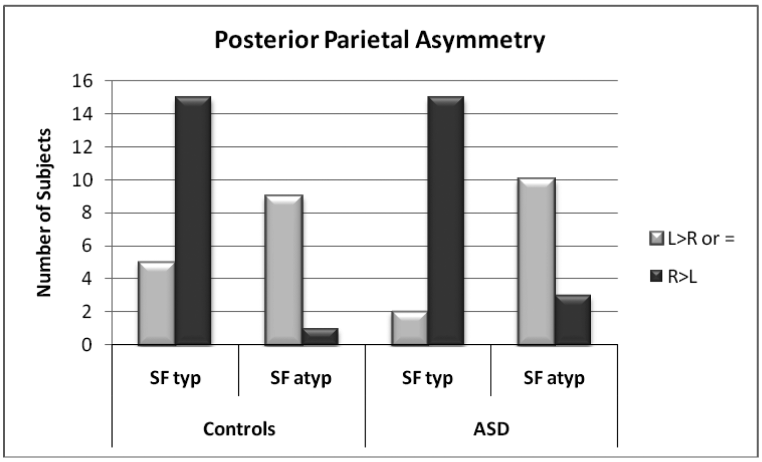

Fig. 5. The number of subjects with rightward posterior parietal asymmetry (typical) and leftward or no asymmetry by SF group and diagnostic group.

rightward asymmetry, while in the atypical SF group, there were more with leftward asymmetry (Fig. 5).

None of the correlations between anatomical ROI measurements was significant, after correction for multiple comparisons.

### *3.4. Relation to language*

After correcting for multiple correlations, there were no significant correlations between any anatomical and behavioral measures.

Dividing the ASD group into low language and average/high language resulted in 10 in the low group and 20 in the average/high language group. The MANOVA examining size did not reveal any significant group or group by hemisphere interaction effects. Similarly, when AQs were examined, there were no significant group differences.

When the ASD sample was divided based on anatomical asymmetry, there were 13 with expected anatomy and 17 with unexpected anatomy. There were no significant differences between these groups in any behavioral measures.

## 4. Discussion

This study was designed to evaluate anatomical differences in SF length and in anterior and posterior parietal lobe volumes in right-handed boys with ASD compared to matched controls, ages 7 to 14 years. There were three major findings in this study. First, the expected morphological asymmetries were found, with a longer left horizontal SF length, a larger left anterior parietal volume (leftward asymmetry) and a larger right posterior parietal volume (rightward asymmetry), in the majority of participants in both groups. Second, there were significant associations between parietal volume and SF configurations, with some subtle differences between the groups, particularly in the anterior parietal region. As predicted, SF length was associated with anterior parietal volume in the typically developing right-handed boys. Specifically, individuals with the typical leftward SF asymmetry had a larger left than right anterior parietal volume and those individuals with the atypical rightward SF asymmetry had a larger right than left substrate. These expected associations differed in the ASD group. Boys with typical SF asymmetry had leftward asymmetry of the anterior parietal, but in the group with atypical SF asymmetry there were more equal numbers of individuals with rightward and leftward anterior parietal asymmetry. Third, within the ASD group, there were no associations between SF length and parietal anatomy with standardized language tests. Each of these results will be discussed in more detail below.

One main objective of this study was to develop a method of examining parietal subregions in reference to SF length. Our results indicate that our method is reliable, with findings consistent with previous length measurements [11,17]. Overall, we found a leftward asymmetry of the SF and anterior parietal region and rightward posterior parietal asymmetry in both groups. Posterior temporo-parietal regions of the brain subserve the asymmetric representation of linguistic, motoric, spatial and emotional processing functions. Anatomical asymmetries of these regions are thought to be related to cortical development and functional lateralization. The longer left extension of the SF is associated with language laterality determined from Wada testing [16] and differs in right and left-handers [14]. The anterior parietal region corresponds to areas shown to be involved in praxis representations (for review [1]). The left hemisphere has been shown to play a predominant role in praxis functions, with lesion studies demonstrating increased rates of apraxia in individuals with left hemisphere damage to this portion of the inferior parietal lobule, compared to those with right hemisphere damage (for review [1]). There is evidence that parietal regions contain mirror neurons, with the left hemisphere involved in mediating the formulation of goal directed actions, including the elemental processes of response selection, programming and initiation that precede the production and execution of an action. This region therefore also plays a role in action understanding and lesions produce impaired tool gestures and gesture recognition/discrimination [52]. In contrast, the right posterior parietal region functions as a topographic map to orient and initiate actions in peri-personal and extra-personal space. Right hemispheric lesions to this region produce spatial neglect [53] and mediate visuomotor spatial actions [9]. Based on these well-established inter-hemispheric differences, we postulate that subtle differences in brain anatomy can inform regarding "neural risks" for specific developmental disorders, like ASD, and may reflect biological vulnerabilities that are in turn likely related to the development of some behavioral anomalies.

Inconsistent with our hypotheses, we did not find statistically significant group differences in size or asymmetry in any of the regions measured. Previous studies have found differences in the parietal lobe in ASD, but results have been variable and only one study has demonstrated an association between parietal anatomy and behavior in ASD [38]. Some studies have found increased volume [30,31], one found increased cortical folding [35], and another found increased cortical folding only in right parietal [36]; some studies have shown increased cortical thickness [32,33], but another study found cortical thinning [34] in ASD compared to controls. These differences are likely due to differences in samples, especially given the heterogeneity of behaviors seen in ASD, as well as differences in methods. Two previous studies have examined parietal volume and found larger parietal lobe size in individuals with ASD [30,31]. These earlier studies, however, differed from the present in several important ways. Palmen and colleagues [30] found increases in gray matter volume, whereas, our method did not segment the tissue into gray and white matter compartments. Piven and colleagues [31] performed measures on the cortical surface and we measured the full volumetric extent of the parietal regions. Furthermore, there are not any studies which have examined anterior and posterior subregions of the parietal lobe, as was done in the current study. Differences in the sample characteristics may also be important. Both previous studies found increased total brain size in the ASD group relative to controls, which we did not find in our sample. Although this was controlled for when examining lobe size in the current study, it indicates potential important differences in the samples. In addition, Piven and colleagues [31]

examined individuals 12 years and older and included some lower-functioning individuals. Our sample was limited to right-handed boys, 7–14 years old, and included high-functioning individuals, with a non-verbal IQ higher than 90. It is unclear if differences in these subregions may be found in lower-functioning individuals with ASD and whether other differences may be partially mediated by sex-linked and handedness factors.

Consistent with the a prior hypothesis, there were significant associations between SF and parietal anatomy in the typically developing boys. As expected, the majority of the typically developing boys had a longer horizontal extension of the SF in the left compared to right hemisphere, with this configuration associated with leftward asymmetry of the anterior parietal ROI and a rightward asymmetry of the posterior parietal ROI. In the atypical SF asymmetry group, there were more individuals with reversed anterior and posterior parietal asymmetry. In the ASD group, similar to controls, most had leftward SF asymmetry associated with leftward anterior and rightward posterior parietal asymmetry. In the atypical SF asymmetry group, however, there were not more individuals with reversed anterior parietal asymmetry, suggesting that in the ASD group there may be slightly more individuals with dissociations between anterior parietal and SF measures. These subgroups, demonstrating anatomical dissociations, warrant further investigation and future studies should examine the relation between cortical region anatomy, in addition to differences in individual ROI size and asymmetry.

In this study, we did not find associations between language and anatomy in the ASD group, as we had predicted. Our measures of language, however, were limited to general receptive and expressive abilities, based on standardized tests. It may be that more specific aspects of language, more related to action understanding or production, would be associated with anatomical measures of the SF and parietal subregions. Functions subserved by posterior perisylvian regions are affected in ASD, such as deficits in language, difficulties with motor planning and mapping other's movements, attentional problems, and superior visuospatial skills. Although there is still controversy and competing theories regarding the central neural defects or vulnerabilities in ASD, a recent review postulated that a multi-modal intervention that would target the MNS could facilitate expressive language development and better social interactions [54], supporting the view that a better understanding of neural structural anomalies and the behavioral correlates in ASD could facilitate the development of targeted therapies to improve the level of functioning of many people. Future studies should examine these functions in conjunction with morphological measures of these posterior perisylvian regions.

There are several limitations to this study. First, this study was limited to high-functioning individuals with ASD and to right-handed males. It would be interesting to examine these anatomical measures in lower-functioning individuals with ASD, as well as to examine the effect of sex and handedness. In addition, although there was a range of language abilities in our sample, inclusion of lower-functioning individuals could result in even more variability in language functions, allowing us to better probe anatomical-behavioral associations. Another limitation is that we only had standardized language measures. More specific aspects of language or other behavioral measures of motor dexterity-skill and cognitive motor ability (i.e. praxis) may reveal interesting relationships between posterior perisylvian anatomy and behavior. Another limitation to this study is that we did not examine gray and white matter separately, which may have revealed differences between the groups. Future studies should take these factors into account in order to enhance our understanding of neural dysfunction in individuals with ASD and the link of anatomical-functional anomalies to behavioral deficits.

## Acknowledgments

This study was supported by a program project grant from the National Institute on Deafness and Other Communication Disorders (U19 DC 03610), which is part of the NICHD/NIDCD funded Collaborative Programs on Excellence in Autism, as well as funding for the GCRC at Boston University School of Medicine (M01-RR0533). We thank all of our research assistants for help in collecting the data and Andrew Silver, Melanee Schuring, Danielle Delosh, and Jeremy Siegal for completing the total hemisphere measurements. We also extend our sincere gratitude to the children and families who participated in this study.
